# Data-Driven Fault Diagnosis for Electric Drives: A Review

**DOI:** 10.3390/s21124024

**Published:** 2021-06-10

**Authors:** David Gonzalez-Jimenez, Jon del-Olmo, Javier Poza, Fernando Garramiola, Patxi Madina

**Affiliations:** Faculty of Engineering, Mondragon Unibertsitatea, 20500 Arrasate-Mondragón, Gipuzkoa, Spain; dgonzalez@mondragon.edu (D.G.-J.); jdelolmo@mondragon.edu (J.d.-O.); fgarramiola@mondragon.edu (F.G.); pmadina@mondragon.edu (P.M.)

**Keywords:** condition monitoring, data-driven, electric drive, fault detection, electric traction, fault diagnosis, machine learning

## Abstract

The need to manufacture more competitive equipment, together with the emergence of the digital technologies from the so-called Industry 4.0, have changed many paradigms of the industrial sector. Presently, the trend has shifted to massively acquire operational data, which can be processed to extract really valuable information with the help of Machine Learning or Deep Learning techniques. As a result, classical Condition Monitoring methodologies, such as model- and signal-based ones are being overcome by data-driven approaches. Therefore, the current paper provides a review of these data-driven active supervision strategies implemented in electric drives for fault detection and diagnosis (FDD). Hence, first, an overview of the main FDD methods is presented. Then, some basic guidelines to implement the Machine Learning workflow on which most data-driven strategies are based, are explained. In addition, finally, the review of scientific articles related to the topic is provided, together with a discussion which tries to identify the main research gaps and opportunities.

## 1. Introduction

Research on Condition Monitoring (CM) and maintenance of electric drives has been a field of activity for decades. Generally, electric drives control software including algorithms, strategies, or routines aimed at actively monitoring their operation by supervising possible system faults. For this purpose, traditionally, model- and signal-based techniques have been used. The former method is based on an analytical redundancy generated by the mathematical model that replicates the operational behaviour of the system under investigation. The latter is based on the analysis of different signals acquired from the real system to identify specific characteristics that indicate anomalies in the equipment. Normally, most of the referred solutions were implemented as on-board or embedded routines in the control software application of the electric drive.

However, the recent advent of Industry 4.0 and the new digital technologies such as Big Data (BD), the Internet of Things (IoT), Cloud Computing (CC) and Artificial Intelligence (AI) have completely changed the paradigm for actively monitoring industrial equipment among which are electric traction systems. An example of the change in this paradigm is the emergent importance of operational data. Owing to the improvement of its availability and the different tools to manage it, presently really valuable information can be extracted from huge amount of datasets. That is why, in many different applications, data-driven strategies have been implemented in order to actively supervise industrial equipment. At the same time, it is important to mention that the aforementioned digital technologies are being offered more as a service than as a product. Therefore, it is no longer justified to increase the cost (memory and computational capacity) of an embedded traction control unit because its health management functionalities, when current AI, CC, and communication technologies allow remote data processing at a much lower cost and greater flexibility.

As an example related to those data-driven fault diagnosis approaches developed in electric drives, some manufacturers already have different IoT-, Cloud-, and Big Data-based health management platforms in the market. Table 1 summarizes the aforementioned main digital platforms in the electric drive sector.

**Figure 1:**
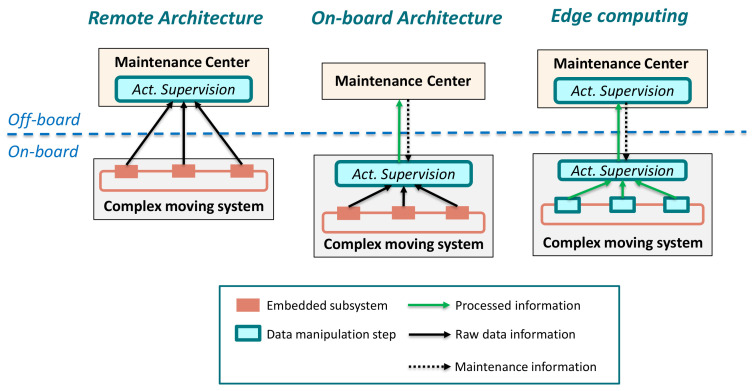
Condition Monitoring and health management architectures applied in electric drives [22].

Although this digital paradigm has become a key point in industry, especially in power traction, due to the increase of e-mobility applications, and power generation, three main challenges have been identified:

- **Adaptation of AI to industry (consumer AI vs. industrial AI).**
  Although AI is well established in the business sector, in the industry still has much to do. The main difference between consumer/business AI and industrial AI is data origin. In the business sector, AI models are fed with human-generated data which is closer to being valuable information than raw data. However, in industry, AI is fed with sensor/machine-generated data. Thus, most of the time, it means working with time-series, which are difficult and more complicated to manage because they need to be processed extensively. More issues related to the characteristics of industrial data were identified in [17,18]:
  – *Lack of faulty data*: Industrial applications are designed not to be prone to failures. Therefore, it is difficult to find data samples to model operation under abnormal events. This lack of faulty samples is an important drawback to develop efficient industrial AI models.
  – *Lack of good quality data*: Data from sensors is noisy, has outliers and contains missing values. Apart from that, in the same system, data of different characteristics and domains (different value ranges, sampling frequencies and origins) are collected. This is the reason of being difficult to manage in comparison to business or consumer datasets.
- **Boosting the collaboration between data analysts and system analysts (Domain knowledge vs. Data Analytics).**
  In [19,20] six key elements (ABCDEF) for Industrial AI are identified: Analytics technology (A), Big data technology (B), Cyber technology (C), Domain Know-how (D), Evidence (E) and Feedback (F). While the first three are usually the domain of a data analyst, the last three are fundamental to ensure the success of any AI strategy. However, many times their presence is not assured. Knowledge of the application is fundamental to understand the system and the problem, knowing which data to collect and understanding the physical meaning of the variables. Thus, there is a need to unify the knowledge and to create communication channels between system and data analysts.
- **Electric drive complexity (Onboard vs. Remote architectures).**
  As mentioned above, electric traction sector is increasingly tending to provide active remote monitoring solutions. For this, the standardized architecture is the one where the onboard equipment only collects and sends raw data. Subsequently, CC platforms carry out data analytics and execution of the supervision models. However, with the increasing data volume generated by sensors, it is clear that sending raw data directly to the cloud is less and less viable. This has reinforced the need for edge-computing. It refers to an architecture in which each subsystem can collect, preprocess, analyze and even execute AI models. As described in Figure 1, there are several alternatives, but clear solution has not been found yet. However, edge-computing is seen as a suitable alternative for transportation applications where assets are geographically distributed, with a large number of fleets and components, high-speed data streams and dynamic environments [2,6,20,21].

**Figure 2:**
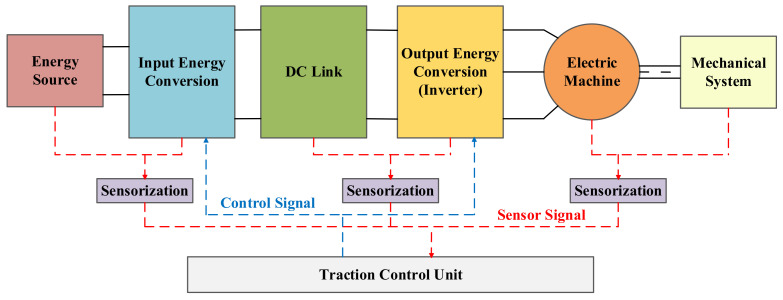
Block diagram of a generic architecture of an electric drive.

All these challenges can be found in the monitoring process of a traction electric drive. Figure 2 shows the most common structure of this system. In general terms, it is composed of an energy source, an input energy conversion step, a DC-Link, an output energy conversion step and an electric machine, these subsystems can be treated as the core of any electric traction application. Furthermore, sensors and the Traction Control Unit (TCU) can be understood as the brain of each application. Moreover, it is worth mentioning that this subsystem structure has been used to organize the scientific documentation review about the topic.

Considering the aforementioned points, this article aims to review the implementation of data-driven active supervision strategies in each of the electric drive architecture subsystems. The main objective is to identify a trend in the use of the different FDD strategies in electric traction applications, focusing on those based on Machine Learning and Deep Learning. The rest of this paper is organized as follows. Section 2 introduces the meaning of actively supervising any industrial equipment, as well as its corresponding standard. Section 3 analyzes the basic theory of the Machine Learning workflow, going a bit on Deep Learning too. Section 4 reviews the applications of data-driven approaches in electric drives. Finally, the discussion about the topic is carried out in Section 5 and concluding remarks are drawn in Section 7.

## 2. Fundamentals of Active Supervision in Electric Drives

Modern industry has developed a trend to design and manufacture equipments with high sophistication, complexity, and capacity that generally increase their Life Cycle Costs (LCC). As a result, and with the aim of producing industrial systems with higher competitiveness in the market, companies have develop greater awareness in key aspects such as Reliability, Availability, Maintainability and Safety, which the four of them compose the RAMS philosophy [23,24,25,26]. These key aspects generally are focused on reducing abnormal operating conditions of industrial systems to avoid their negative consequences [27,28]. Therefore, it is interesting to actively supervise industrial equipment, in order to control their operation and perform anomaly detection and identification tasks, or even predict the health status of their components.

**Figure 3:**
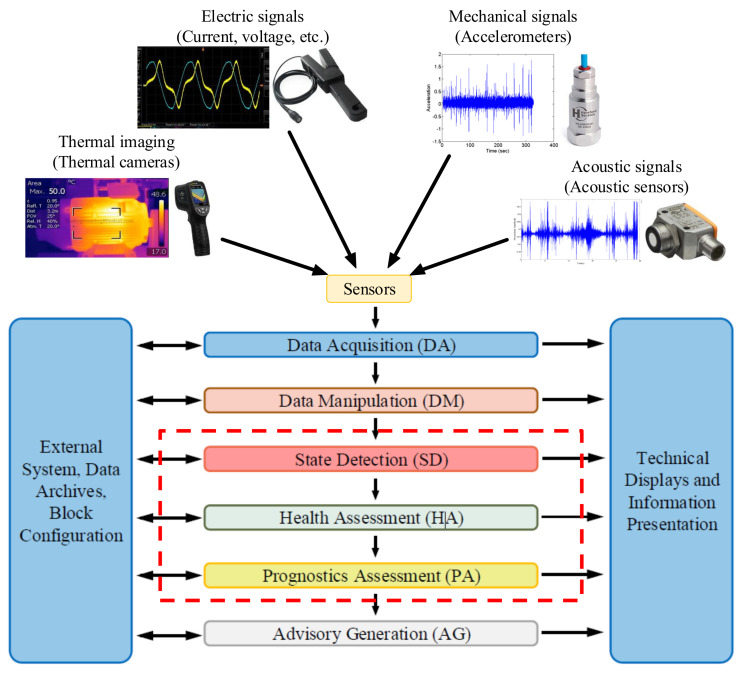
Block diagram of the ISO 13374 Condition Monitoring standard architecture [30].

Supervision is understood as the set of actions executed with the purpose of ensuring the correct operation of any system [29]. Presently, there are different standards, such as ISO 13374 [30,31,32,33] or CRISP-DM [34,35], which describes a modular architecture to supervise and monitor industrial equipment. Concretely, Figure 3 shows the block diagram presented in the ISO standard.

It can be said that every active supervision strategy will contain three common blocks which namely are the Data Acquisition (DA), Data Manipulation (DM) and the Advisory Generation (AG). In other words, some kind of information should be acquired from somewhere, for example sensors, transducers, etc. Consequently, this information usually should be preprocessed in order to optimize the knowledge value. In addition, of course, at the end of this process, certain information must be delivered to the system maintenance personnel, designers, users, etc. However, the blocks ((SD), (HA) and (PA)) inside the dotted red box in Figure 3 depend, to a large extent, on the supervision methodology chosen. This means that the modular architecture, and thus the active supervision strategy, can be limited only to Detection tasks (check if a fault has happened in the system, via alarms). It can be partially extended until Diagnosis tasks (know what and where has happened in the system). Or even, it can be extended totally until Prognostics levels (identify what will happen in the near future, the so-called Remaining Useful Life (RUL) estimation).

**Figure 4:**
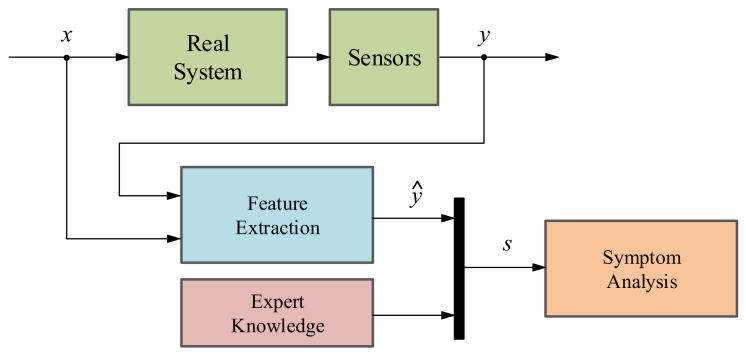
Schematic of the signal-based fault detection and diagnosis methodology.

**Figure 5:**
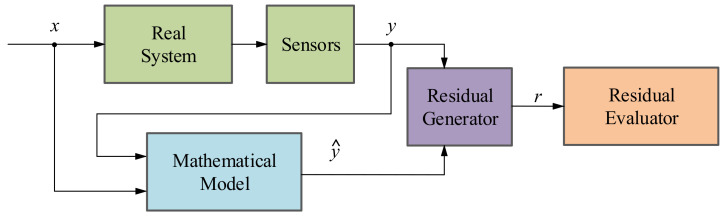
Schematic of the model-based fault detection and diagnosis methodology.

**Figure 6:**
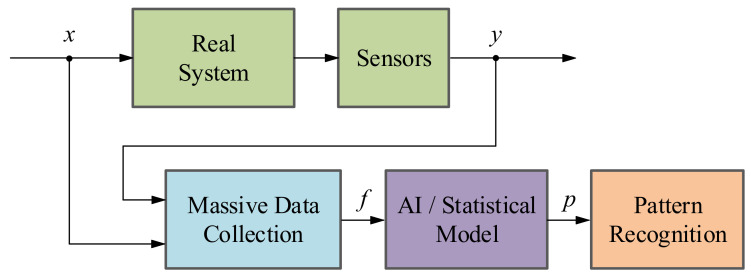
Schematic of the data-driven fault detection and diagnosis methodology.

There are four main methodologies to implement these last functionalities:

- *Signal-based methodology*: The main objective of a SB method is to analyze fault symptoms (*s*) in the real signals acquired from the systems under investigation such as current, voltage, vibrations, etc. In a nominal state, the acquired signals correspond to certain frequency, amplitude and ripple features (*ŷ*) whereas in faulty states these indicators differ from the nominal ones [36,37]. That is why with the help of expert knowledge, it can be identified whether a fault is presented or not in the system (see Figure 4). It is important to know that the existing SB methods can be categorized depending on their feature typologies: Time domain techniques, frequency domain techniques and time/frequency domain techniques [38].
- *Model-based methodology*: This method aims at generating an analytical redundancy through a mathematical model that replicates the physical behaviour of the real system. The MB strategy executes the analytical model in real-time, in parallel to the real system and with the same measured inputs. After that, the mathematical model output (*ŷ*) is compared with the output of the real system (*y*), generating residual signals (*r*) which will determine if there is a fault in the system using a residual evaluator [36], as it can be seen in Figure 5. According to [36,39,40], there are three different model-based approaches: the parameter estimation approach, the parity space approach and the observer-based approach.
- *Data-driven methodology*: The basis of DD methods is to take advantage of large amount of historic datasets acquired from the system under investigation by means of Machine Learning or advanced statistical models [36,41]. These algorithms learn from data in order to discover hidden patterns (*p*) represented in the information redundancy among the system variables (see Figure 6). It can be said that this approach is the recent alternative for active supervision in those systems, which are too complex to have an explicit analytic model or signal symptoms of faulty behaviour. It is worth mentioning that the information redundancy added to AI techniques make possible to build a complete CM platform, from data acquisition (DA) to prognostics assessment (PA) levels.
- *Hybrid methodology*: As the aforementioned active supervision techniques have their pros and cons, a new trend has emerged, which tries to integrate together these complementary methods in order to achieve a better performance [41,42]. It is commonly agreed that hybrid schemes would provide better solutions to a complex system. In other words, hybrid CM methods aim to enhance the supervision results by leveraging the advantages and avoiding the limitations of their consisting approaches.

Table 2 collects the most important advantages and disadvantages of the different aforementioned methodologies.

To sum up, on the one hand, the limitations of SB and MB methodologies are the excessive requirement of expert knowledge and their limitations to implement all the functionalities from the ISO architecture. On the other hand, the main drawback of the data-driven method is the availability of the data itself. However, the improvement of the data manipulation and communications, as well as the trend of acquiring data in every company make data-driven strategies a hot topic in present research.

## 3. Fundamentals of the Machine Learning Workflow

This section provides an explanation of the standardized workflow to develop a data-driven fault detection and diagnostic (FDD) strategy based on Machine Learning algorithms, for actively supervising electric drives.

**Figure 7:**
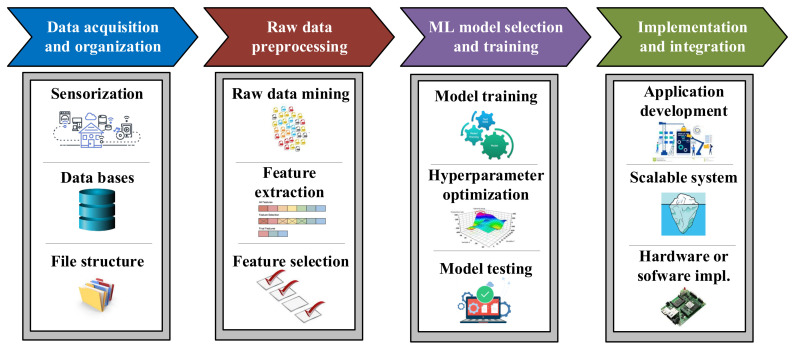
Machine Learning standardized workflow [43].

Although sometimes it is thought that implementing these solutions consists only of selecting and optimizing a Machine Learning algorithm, many other operations have to be carried out in order to develop efficient approaches. As Figure 7 shows, this methodology can be split in four main stages, which namely are the acquisition and raw data organization steps, the raw data preprocessing step, the Machine Learning model design and the implementation and integration in the application [43,44,45].

### 3.1. Acquisition and Raw Data Organization

In this first stage, the massive raw data acquisition is performed, as well as its saving and organization in a structured way. This step is very important because the quality and the quantity of the data gathered will directly determine how good the FDD model can be. The raw data acquisition involves reading and saving data from different sources, not only from online streaming sensors directly installed in the system under investigation, but also from offline databases such as historic datasets from local or cloud data servers.

It is worth mentioning that in order to build a ML-based solution, which generalizes every health condition of an industrial equipment, both healthy and faulty data must be acquired. However, as mentioned before, this is an important problem that DD methodology must deal with. In most of the industrial applications, the observed data volume for nominal or healthy operations far exceeds the observed data volume for an anomalous or faulty status, which will cause great difficulties in conducting the model training. Owing to the unbalanced datasets, it is especially necessary to acquire more data under fault status scenarios or apply data augmentation techniques, which will make the entire dataset more balanced [18]. A well-established example of data augmentation is synthetic data generated from simulation via the so-called Digital Twins [46,47].

Likewise, it is not only important to collect such data. Where and how to store it is also of crucial importance. The standardized storage place has usually been local servers (Local databases), but in recent decades the tendency is to use services in the cloud (cloud databases) because of their improvement and flexibility. Moreover, the data should be stored in a structured way in order to be more efficiently accessible.

### 3.2. Raw Data Preprocessing

After acquiring and organising the raw data, the next step is preprocessing it. That means, data manipulation and transformation for consumption in Machine Learning algorithms. In general, this step can be divided into two levels of preprocessing. On the one hand, the general raw data preprocessing and on the other hand, the specific feature preprocessing or Feature Engineering.

- *General raw data preprocessing*: This level of preprocessing involves data cleaning, which is all about filtering messy data, detecting outliers and missing values, and applying normalization [48,49] and even segmentation [50,51]. Furthermore, it is important to know that data exploration is a technique that will help general preprocessing by combination of manual methods and automated tools such as data visualizations, charts, etc. to understand what is in a dataset. After refining the raw dataset, the volume of data will be considerably smaller. This makes easier to manage it during the Feature Engineering step.
- *Feature Engineering*: Once the raw data have been reduced and cleaned, Feature Engineering should be applied in order to modify the dataset into appropriate information for feeding the ML algorithm. Particularly, Feature Engineering can be split into two main tasks, Feature Extraction (FE) and Feature Selection (FS).
  The main objective of Feature Extraction is to transform raw data into numerical features that can be processed by the ML algorithm, while preserving the information in the original dataset. ML algorithms do not always work so well on raw data, that is why extracting new numerical features from historic dataset variables yields better results. This task performance is directly related to domain expertise and solid understanding of the data, that is why expert knowledge is really required. Feature Extraction can be accomplished manually, via time, frequency and time/frequency domains transformations such as mean, maximum/minimum, standard deviation, kurtosis, as well as, more advance domain transformations such as Fast Fourier Transform (FFT) or even Wavelet and Short Time Fourier Transform (STFT) approaches. At the same time, FE can be also performed automatically using specialized algorithms such as Linear Discriminant Analysis (LDA), Principal Component Analysis (PCA), or Independent Component Analysis (ICA) [52,53,54].
  Usually, a huge number of features can be obtained from the FE stage. Unfortunately, not all features are meaningful and contain hidden patterns about the equipment under investigation. That is why some of them are redundant or irrelevant. These features should be removed by means of Feature Selection techniques [52]. The main objectives of applying these methods are the accuracy improvement in the ML model, the overfitting risk reduction of the algorithm, and a considerable speed up in the training step. When speaking about overfitting, it means that a trained model corresponds too closely to a particular dataset, which may therefore fail to fit additional data or predict future observations reliably [55,56]. Therefore, this second task of Feature Engineering tries to rank the importance of the extracted features in the dataset by applying certain evaluation criteria, while discarding less important variables. FS mechanisms are divided in three groups: Wrapper, Filter, and Embedded methods [57].

### 3.3. Machine Learning Model Design

Once the most important features have been selected, it is time to choose the most appropriate Machine Learning topology and algorithm to build the active supervision strategy.

Machine Learning is a subset of AI which enables computers to act and make data-driven decisions to carry out certain tasks. Its main applications can be summarized in collaborative filtering, automatic translation, speech recognition, face identification, as well as different fault diagnosis and maintenance tasks [58]. The core of these data-driven methods are algorithms that are designed in a way that they can learn and improve over time when exposed to new data [59,60,61].

**Figure 8:**
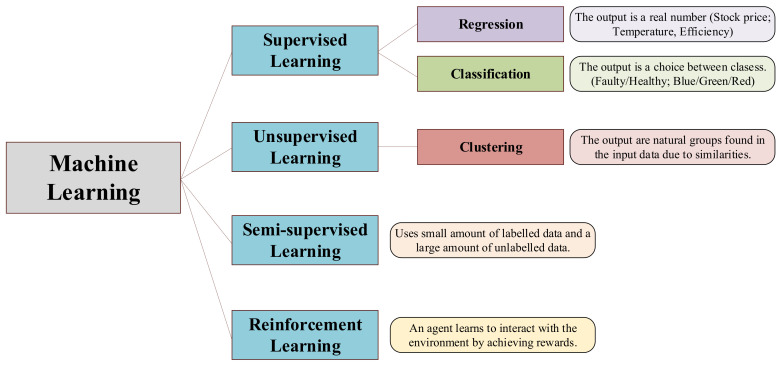
Machine Learning algorithms topology.

ML algorithms can deal with different problem topologies and applications. The typical ML classification shown in scientific publications such as [60,61,62,63,64] is the one presented in Figure 8.

**Figure 9:**
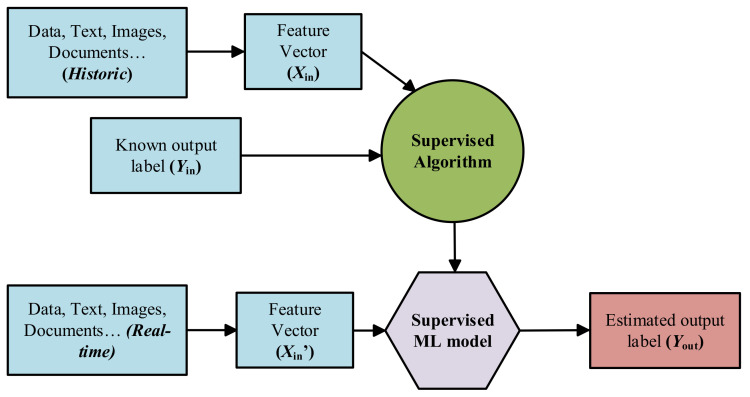
Schematic block-diagram of the supervised ML approach.

**Figure 10:**
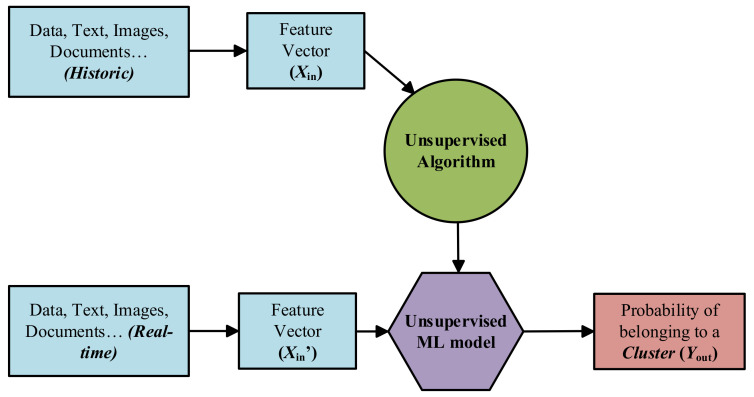
Schematic block-diagram of the unsupervised ML approach.

- *Supervised Learning*: Supervised ML algorithms are supported by an established set of data and a certain understanding of how that data is organised. That is to say, data samples have assigned labels that define the meaning of the input/output relationship [65], as it can be seen in Figure 9.
  However, the most challenging problems in supervised models is that they require labelled data. One has to spend time labelling the samples, which is expensive and time-consuming.
- *Unsupervised Learning*: Unsupervised ML algorithms arise when the problem contains a large amount of unlabelled historic data. That means that it only contains the features subset, no label characteristic is available [65] (see Figure 10). Thus, understanding the meaning behind this problem requires algorithms that can find and classify data instances into groups by their similarities based on distance or statistic metric [61,66].
  Clustering is the most used Unsupervised approach. With it, objects or samples with similar parameters or characteristics are grouped together in “clusters”.
- *Semi-supervised Learning*: Most of the application domain suffers from not having sufficient labelled data whereas unlabelled data are available cheaply. Semi-supervised Learning addresses this problem and act as a half way between supervised and unsupervised learning [67,68]. Normally, first, these type of algorithms use the limited set of labelled samples to train themselves, resulting in “partially trained” models. After that, the partially trained models label the unlabelled dataset. In addition, finally, the pseudo-labelled and labelled datasets are combined, in order to train a new algorithm that joins both the descriptive and predictive aspects of supervised and unsupervised learning.

**Figure 11:**
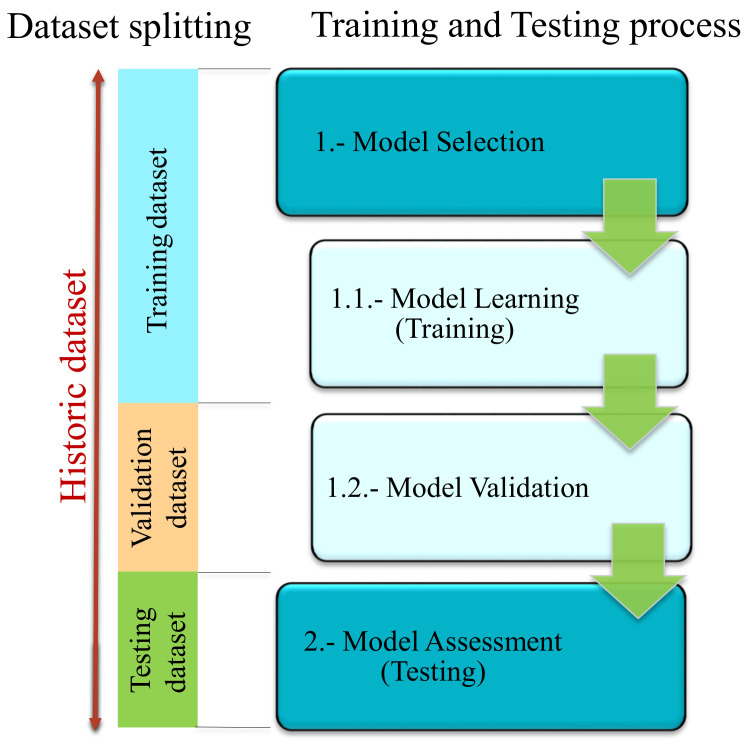
Training/Testing process for a ML algorithm.

As said before, in this third step of the workflow, the Machine Learning topology should be selected and the algorithm should be trained and validated in order to leave it ready to integrate into the required application [62,69,70]. For that, the specific procedure shown in Figure 11 should be followed [71].

In the Model Selection phase, an empirical comparison of algorithms of the same topology is performed and the approach with best results is selected. This phase is divided into two sub-phases. On the one hand, in the first sub-phase (Model Learning), algorithms of similar characteristics (supervised, unsupervised, etc.) are trained with the training sub-dataset. This means adjusting their internal parameters during the learning phase. On the other hand, in the second sub-phase (Model Validation), hyperparameters are optimized and the different algorithms are validated with the validation sub-dataset. Validation means, evaluating algorithms performance by different criteria. The most common indicators are the Confusion Matrix for classification problems and the Root Mean Square Error (RMSE) for regression approaches [72].

Finally, in the Model Assessment phase the trained and selected algorithm is checked in a real environment under new unseen data, the so-called testing sub-dataset. If this last evaluation is positive, the ML model will be ready to be implemented in the application.

In addition, it is important to mention that the most efficient way to carry out this training and testing process is to use independent sub-datasets at each stage. That means that a historic dataset achieved from preprocessing step of the workflow should be split into three sub-datasets, which are training, validation and testing sub-datasets, as was said before (see Figure 11). Normally, between 60–70% of the historic dataset is saved for training tasks. The rest 20–30% is split in two equal parts for creating the Validation and Testing sub-datasets.

### 3.4. Implementation and Integration in the Application

In this last step of the ML workflow, trained and validated models are integrated into company pipelines. In the case of a data-driven active supervision strategy, this step means that ML algorithms are integrated in the CM structure from Figure 3. However, several tasks need to be performed before a data-driven solution is fully operational. Some of the aspects to be taken into account are:

- The ML workflow may not be developed using industrial tools. It is common to use research or academic tools (such as Matlab) in the training and validation phases. Hence, during the deployment, algorithms need to be adapted to different platforms.
- Following the previous point, visualization tools need to be developed. In the design phases, specialized tools are used to analyze in detail the performance and operation of the algorithms.
- The deployment architecture must be selected between the solutions given in Figure 1.

## 4. Data-Driven Active Supervision Strategies in Electric Drives

In this section, a review of scientific documentation focused on data-driven active supervision techniques applied in electric traction systems is presented. As a way of covering as many publications as possible and give consistency to the review, a structured material collection was carried out. Therefore, this review is defined by the next points:

- The collected material is composed of scientific papers, from many data bases such as Web of Science, Scopus, Google Scholar, etc. in which the main area of study matches with data-driven fault detection, diagnosis and prognosis approaches performed in electric drives.
- The searching has been limited by dates, only documentation published between 2010 and the present has been taken into account.
- The publications have been classified by application type, component under investigation, ML type, ML algorithm, preprocessing techniques and data sources.
- Finally, the literature has been organized depending on the electric drive subsystem they belong to, taking as reference the general architecture defined in Figure 2.

Therefore, it is important to take into account that the aim of this review is to analyze the present studies and research about this topic, in order to identify the main challenges around it, to be able to define new investigations gaps for future developments.

### 4.1. Energy Source

In electric drives, the energy source can be based on different topologies such as batteries, electric grid, catenaries, super/ultra-capacitors, etc. which depend mainly on the application.

In the railway sector, catenaries or overhead contact lines (OCL) are treated as part of the infrastructure section. This means that this component should be considered out of the electric traction system of a rolling stock itself. However, owing to its relevance in the performance of the system and its continuous interaction with the pantograph, they have been taken into account in this review.

To start with, ref. [73] reviews the state of practice and the state of the art of Prognostics and Health Management (PHM) strategies for OCL systems. Key sensors, monitoring parameters, state detection algorithms, diagnostics approaches and prognostics models are reviewed. Additionally, future challenges and technical needs are highlighted, such as the need for standardizing sensors, improving in data storage platforms, and boosting hybrid FDD strategies.

In the case of ref. [74], a brief comparison between the traditional methods used to detect faults in catenaries and the more sophisticated ones is presented. It remarks that traditional ones were carried out through foot inspections and inspection trolleys fitted with cameras, proving to be inefficient and time consuming when analysing the data. However, with the advance of AI and new sensing techniques, smart supervision systems based on ML techniques have been implemented. As an example, the paper analyzes the interaction between the pantograph and contact wire (CW) by using accelerometers and non-contact infrared thermometers. The acquired field data are transmitted to the cloud for preprocessing and storage. Moreover, an unsupervised learning method based on the *k*-Means clustering algorithm is built in Matlab, which reaches satisfactory results.

Furthermore, ref. [75] focuses on catenary-pantograph interactions, especially in electric arcs. The general block diagram of the designed data-driven active supervision strategy is based on three SVM classifiers fed with current and voltage signals from pantographs, working as an ensemble method. At the end, a fuzzy integral technique is used to synthesize the results obtained by the individual classifiers reaching 95.64% of accuracy in electric arcs’ detection tasks.

When focusing only in the pantograph device, it is clear that one of the main problematic events is the carbon contact strip wearing. An example of this event analysis is ref. [76], which develops a RUL prediction of the pantograph carbon contact strip (PCCS) via linear regression combined model. For that, wear data from Guangzhou Metro are acquired and preprocessed, reaching acceptable predictive properties.

Eventually, in terms of energy storage systems (ESS) such as batteries and super/ultra-capacitors, plenty of studies have been carried out in levels of fault detection and diagnostic or even in prognostics ones. On the one hand, as reviews such as [77,78] say, fault diagnosis of batteries is an important task in the battery management system (BMS). It is responsible for detecting early faults and providing control actions to minimize their effects, to ensure the safe and reliable operation of the ESS. On the other hand, research such as in refs. [79,80,81] concludes that RUL prediction of batteries has become the hot topic in electronic PHM, because it is helpful to reduce failure rates and maintenance costs.

However, an exhaustive analysis of the existing alternatives is outside the scope of this review, since the ESS sector is large enough to warrant an independent analysis.

### 4.2. DC Link

The DC link subsystem makes the union between the input and the output energy conversion subsystems using a bus capacitor bench. Other elements can be added to this capacitor bench to actively control the flow of energy, such as the crowbar and the grounding circuit.

In the case of the crowbar, the influence of AI is not usual. No scientific papers have been found concerning maintenance strategies of this protection element. The main reason for that can be the simplicity and the discontinued use of the component, since it is only used in braking mode and not in every application.

In respect to the grounding circuit, a single paper was found [82]. The research goes over a data-driven FDI method to avoid grounding faults and consequent short circuits. In this case, the Canonical Correlation Analysis (CCA) supervised algorithm is applied for fault detection, and then fault isolation and visualization techniques are proposed based on DC link current and voltage data from a test-bench.

However, as review [83] transmits, a large number of scientific publications studied CM strategies in capacitor benches during last two decades. Most of the research is based on signal-based methodologies. Taking refs. [84,85,86] as example, the main strategy in this approach is to detect changes in the equivalent series resistance (ESR) and in the capacitance of the component via the analysis of voltage and current measurements, in order to detect the life-cycle status and permit preventive maintenance of the component.

Nevertheless, a tendency can be observed from 2015 until now, where research raised attention in data-driven active approaches. For example, a data-driven method for capacitors based on an Artificial Neural Network (ANN) algorithm is proposed in ref. [87]. It is applied to a back-to-back converter case study to estimate the capacitance value change of the DC-link capacitor. Data for training and validating the model is achieved from simulations in Matlab/Simulink software.

The case of ref. [88] presents a fault detection and identification method for the capacitor ageing faults in DC filters of power converters. This strategy is based on the adaptive neuro-fuzzy inference system (ANFIS) algorithm. The inputs to this model are input voltage of the converter, as well as the voltages across the DC filters. The output of the ANFIS unit is used as an index to identify the capacitor ageing fault. Then, it locates the fault within the two DC filters installed in the power converter. Another recent example is ref. [89], where an online failure detection method for a DC-link electrolytic capacitor in a converter, using a Support Vector Regression technique, was proposed.

Table 3 gathers the analyzed papers concerning data-driven fault detection and diagnosis models applied over the DC link subsystem.

### 4.3. Energy Conversion Step

In the energy conversion steps (input and output), the power converters are normally based on power semiconductors, which are exposed to high thermal and electrical stress in electric drive applications. Repeated impact of energy during the IGBT’s or Diode’s switching and blocking makes the power converter more vulnerable to failures, such as open-circuit (OC) and short-circuit (SC). Therefore, supervision of this subsystem has been studied for years.

Until 2010, signal-based and model-based techniques were the most applied methodologies. On the one hand, signal-based methods such as current trajectory and instantaneous frequency analysis, considered in refs. [90,91], were applied because of their simplicity and low computational load in detection supervision levels. On the other hand, the model-based techniques were more precise and easier to implement in diagnostic tasks after the mathematical model generation [92]. Nevertheless, as the complexity of the systems have increased, the work required in calculating these new mathematical models have not been feasible.

That is why, from 2010 to the present, data-driven active supervision strategies have been investigated because of the improvement of data accessibility, storage and computational capacity. One of the first works implementing data-driven methods in power electronics semiconductors supervision is ref. [93], which with the help of model-based simulations, detects and identifies IGBT OC faults from voltages and current signals via ANN. Additionally, supported by simulations in Matlab, ref. [94] develops an IGBT OC diagnosis model based on a SVM. As peculiarity, in this research the current signals are preprocessed with wavelet transform in order to achieve new input features to the model.

In ref. [95] not only the OC fault mode is analyzed, but also SC faults are distinguished via an ANN algorithm. In this case, voltage, current and torque variables are preprocessed to calculate new statistical features such as maximum, minimum, standard deviations, etc.

Furthermore, in ref. [96], a basic test-bench with an electric drive attached to a microcontroller is built. The main objective of this test-bench is the massive data generation (healthy and faulty). In this research, new features are extracted with FFT from output inverter voltage, and then the most relevant ones are selected with PCA method. Finally, a Bayesian Network is developed to diagnose OC faults.

However, research developed from 2016 until now focused more on Deep Learning approaches. As an example, ref. [97] questions both model-based and basic ML architectures because they need to build complex mathematical models or extract features from sensor signals manually. To solve these problems, the paper proposed a new DL method for IGBT OC fault diagnosis based on a Convolutional Neural Network. This end-to-end algorithm extracts comprehensive information from converting current signals into pixelated images. It is worth mentioning that faulty and healthy data for training the CNN is acquired from simulations in Matlab/Simulink.

Another example of DL application is ref. [98], where a fault identification in traction inverter based on Deep Wavelet Neural Network (DWNN) and Deep Support Vector Machine was suggested. The method uses the DWNN to automatically mine and compress hidden fault information from the simulated current signals and after that Deep Support Vector Machines (DSVM) are trained to integrate the recognition results.

Similar to previous examples, in ref. [99], a robust fault diagnosis strategy for open switch faults isolation in a five-phase conventional inverter was designed. An adaptive Self-Recurrent Wavelet Neural Network (SRWNN) as a non-linear system identifier provides the fault detection scenario. Then, it is followed by a classifier to locate the fault. Discriminant Analysis and SVM have been implemented to identify the fault location. In this research, the proposed method was also evaluated by experimental results obtained from a lab prototype.

To sum up, all the analyzed scientific papers referring Data-driven FDD techniques in inverters of electric drive applications are shown in Table 4.

### 4.4. Electric Machine

In the case of the electric machine subsystem, plenty of scientific publications have been found. This can be for many reasons. On the one hand, the electric machine can be categorised as the nucleus of electric drive application, because it is the device which generates the motion. On the other hand, it is one of the most expensive and complex part among all the components.

Traditionally, signal-based methods have had a wide application in Condition Monitoring for electric machines, both in electrical and mechanical faulty events. Reviews [108,109,110], and PhD thesis [111] summarized the different applied methodologies. Moreover, model-based diagnostic methods have been also used simultaneously with signal-based method. In this case, reviews such as [112,113] make a general mapping of this strategy in electric machines, and scientific applications such as [114,115,116,117] develop different model-based methods.

Even so, concerning the last decade, data-driven techniques were implemented to assist in active supervision tasks. As an example from 2010, ref. [118] performed the complete workflow of a Machine Learning based active supervision methodology for electric machines. It classified winding inter-turn SC and rotor eccentricity faults based on a Multi-Layer Perceptron (MLP). Later, ref. [119] analyzed electric machine health status not in steady state, but in speed transient state. In this case, stator current signals have been preprocessed by wavelet transform and PCA algorithm to achieve new input features for the Decision Tree model.

However, not only electric signals are analyzed in electric machine diagnosis, refs. [112,120] analyzed stator and rotor faults via vibration signals from accelerometers installed in test-benches. The research compared *k* Nearest Neighbours, MLP and Radial Basis Function (RBF) algorithms performance after preprocessing vibration signals with discrete wavelet transform (DWT) and genetic algorithm (GA). The latter calculates statistical features and principal components from vibration signals to feed an ANN algorithm. Furthermore, research where acoustic signals [121,122] or even thermal imaging [123] are analyzed, can be found in the bibliography.

Although every research shown until now reached diagnosis classification accuracies above 90%, they depended strongly on expert knowledge, especially in preprocessing steps such as feature selection and extraction. Thus, from 2016 to the present, Deep Learning workflow has been studied in different publications. As mentioned before, Deep Learning models substitute the time-consuming preprocessing steps by end-to-end algorithms which work directly with quasi-raw data, accelerating the workflow and approaching to real-time maintenance strategies. For example, ref. [124] trained a Deep Belief Network (DBN) with vibration data from an electric machine. This algorithm is a probabilistic generative model formed by Restrictive Boltzmann Machines (RBM), which can model high-dimensional and non-linear data via multiple layers, thus can reduce training error and improve classification accuracy.

In ref. [125], stator and rotor faults were analyzed with CNNs. It is important to mention that in this case, CNN training periods are reduced because the Transfer Learning approach has been applied. This recent philosophy takes advantage of parameters and hyperparameters from previous trained CNN of similar applications. Refs. [126,127,128] are other examples were end-to-end algorithms were used to actively supervise electric machines, Sparse Autoencoders combined with SVM (SAE-SVM), CNN and DNN respectively.

Table 5 collects the papers which perform a data-driven fault detection and diagnosis methodology in electric machine.

### 4.5. Mechanical Subsystem

In this scientific documentation review, the mechanical subsystem is the one that concentrates the highest amount of scientific publications. Specifically, among all the components that compose the subsystem, bearings are the most analyzed elements. For example, reviews such as [145,146,147,148] affirm that this vulnerable element has been studied for a long time. In general terms, they analyzde both ML and DL approaches for detection and diagnostics tasks, as well as, for prognostics tasks applied in bearings.

Based on the carried out analysis, it can be seen that from 2010 to 2015, the ML workflow was the basis of the data-driven active supervision techniques applied in bearings. Next publications are examples of this period of time. Ref. [149] develops a FDD strategy based on a SVM classifier applied in bearings of a three-phase induction motor. In this case, data are acquired from accelerometers installed in a test-bench, and preprocessed via Continuous WT to improve the training dataset quality.

Moreover, ref. [150] proposed a binary classifier based on an ANN. This algorithm is tested with experimental data obtained via the phase current measurements when the machine is in healthy state and having cracked bearing fault. It concludes that the success of a classifier depends very much on the effectiveness of the extracted features more than in the algorithm itself.

Furthermore, in ref. [151] a monitoring scheme applied to diagnose local defects, raceway faults and also, distributed anomalies in bearings was presented. For that, first, the most significant statistical time domain features are computed from vibration signals. Then, in order to comprehend and visualize features behaviour better, Curvilinear Component Analysis, a non-linear preprocessing technique was applied. Finally, a Hierarchical Neural Network structure was used to perform the classification stage between classes.

However, from 2015 until now, DL architectures have been the most popular in bearing active supervision strategies. Clear examples of this change are next scientific publications. Refs. [152,153] researched the design of CNN-based end-to-end methods that take raw signals as inputs. The only difference between both publications is that the former uses as input image the frequency spectrum of the vibration time-series, whereas the latter uses the STFT of those vibrations signals. It is important to mention, that in both cases the dataset has been taken from a public dataset provided by Case Western Reserve University. It consists of vibration signals collected at 12 kHz or 48 kHz for normal and damaged bearings with single-point defects under four different motor loads.

In the case of ref. [154], it faces the noise drawback in vibration signal from bearings to develop an effective data-driven FDD methodology. It uses the Deep Autoencoder algorithm to denoize input signals before putting them into the Neural classifier. This denoizing step improves classification accuracy of bearing faults until values above 99%.

At the same time, and taking advantage of the DL’s strengths, strategies based on acoustic signals or thermal imaging have been applied for diagnosing faults in the mechanical subsystem of electric drives. As an example, on the one hand, ref. [155] proposes a CNN-based classification method for diagnosing bearing faults under variable shaft speeds using acoustic signals. These signals are represented by spectrograms to obtain as much information as possible in the time–frequency domain. On the other hand, ref. [156] worked in a new framework based on small labelled infrared thermal images used to train CNNs.

Apart from fault detection and diagnosis strategies, prognostics techniques to compute the RUL of bearings are also being developed. As an example, ref. [157] dealt with the problem of CBM applied to the predictive maintenance of train axle bearings based on multi-sensors data collection, with the purpose of maximizing their RUL. For that, SVR algorithm is trained and tested with real data. Additionally, ref. [158] worked on a data-driven approach for the RUL estimation of rolling bearings based on a SVR algorithm. In this case, multiple statistical features in time and frequency domain are extracted from the run-to-failure experiments by the PRONOSTIA public dataset provided by the FEMTO-ST institute in France.

To end with this subsystem review, it is important to mention that apart from bearings, data-driven active supervision strategies also are focused on the gearbox. Some examples are refs. [159,160], which based their diagnosis abilities in CNN models trained with vibration signals acquired from test-benches.

Table 6 collects the most important papers concerning data-driven active supervision strategies applied in mechanical subsystem.

### 4.6. Sensors

Although the function of sensors is very wide, the majority of the reviewed research analyze sensors which are used for control operations. For example, in ref. [184] a new data-driven incipient fault detector methodology was proposed via Neural Network algorithms for phase current, speed and DC link voltage sensors. It incorporates preprocessing algorithms such as PCA or Kullback–Leibler divergence (KLD) to extract important information from the acquired data.

Furthermore, ref. [185] developed a SVM based fault detection and diagnosis strategy applied in High-Speed Trains applications. On it, the fault detectability of data from a test-bench is improved via PCA preprocessing methods. Additionally, studies such as [186,187] develop generic faults diagnosis strategies for sensors, based on Extreme Learning Machine and *k*NN algorithms, respectively. Both studies are focused on current and speed signal measurements acquired from simulations and test-benches.

However, few studies focused on sensors used in protection or monitoring tasks. For example, ref. [188] demonstrated that ML techniques such as *k*NN can be used to classify generic sensor faults from a strain gauge used in aviation application. It is worth mentioning that it is the only research found that tests the ML model against field data. Moreover, ref. [189] analyzed temperature sensor performance installed in a converter package from a traction application. It develops a SVM based classifier to detect and diagnose faults such as erratic, drift, hard-over, spike, and stuck by inserting them in a simulation run in Matlab. In this case, preprocessing tasks have been carried out in order to extract features in time domain via basic statistics methods.

Table 7 summarizes papers which perform a ML based data-driven fault detection and diagnosis methodology.

## 5. Discussion

This section presents the main discussion of the review of the ML- and DL-based solutions for actively supervise electric drives. These results have been divided in points in order to facilitate their understanding.

**Figure 12:**
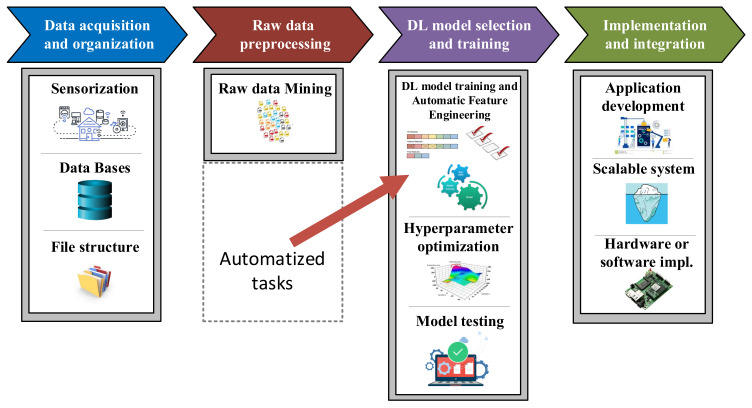
Deep Learning workflow.

**Figure 13:**
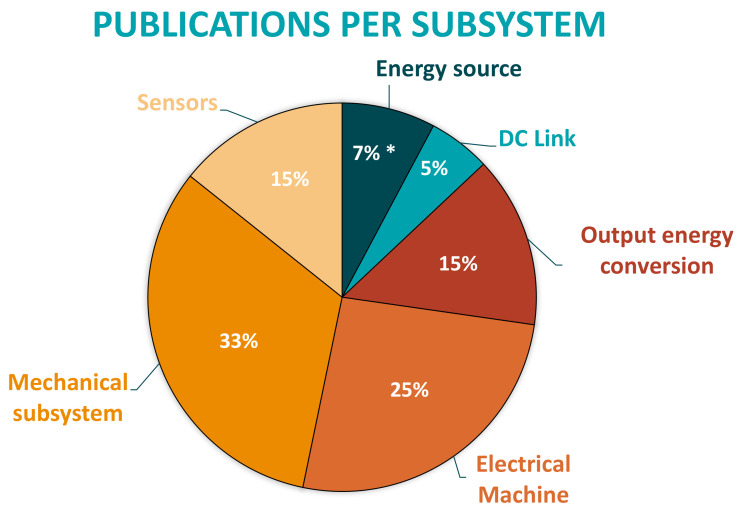
Percentage of developed research per subsystem of the electric drive architecture. * Batteries and Super/Ultra-capacitors have not been analyzed because they have potential to be studied independently.

**Figure 14:**
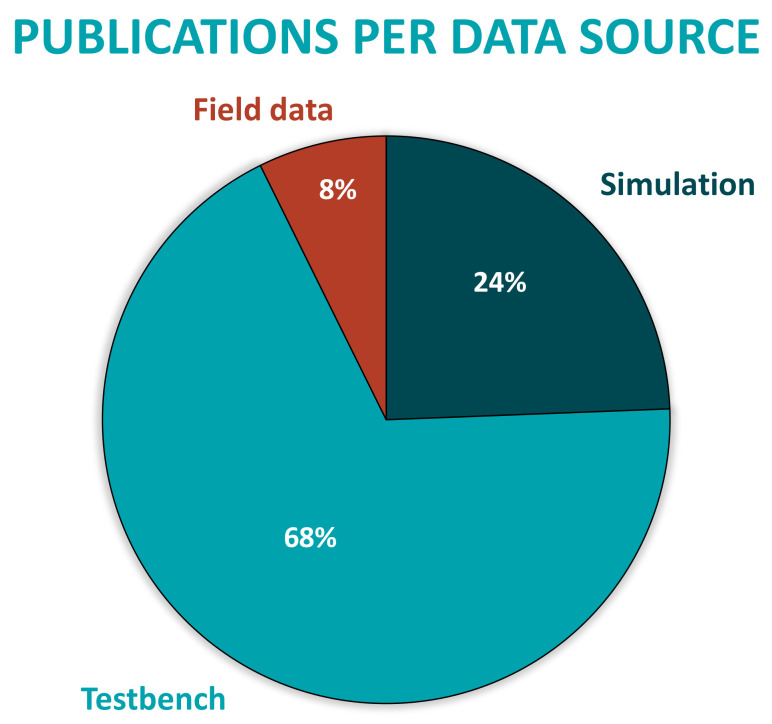
Percentage of developed research per data source.

- First, taken the aforementioned bibliography into account, it is important to understand the tendency of the different FDD methodologies applied in electric drives during last decades, in order to understand the background and the future lines about this topic. Therefore, as a result, this review shows that until 2010, model- and signal-based methods were the most applied strategies by researchers. However, due to the development of the Industry 4.0, as well as the enhancement in the accessibility to large amount of datasets, from 2010 to the present, solutions based on data-driven methods has been the most studied and developed. Compared with the classical techniques, DD active supervision strategies have a greater scope, as they can embrace more functional levels of ISO 13374. While MB or SB techniques cover until the State Detection or even Health Assessment level, data-driven ones can also help at Prognostics Assessment and Advisory Generation tasks.
- Furthermore, it is important to know that these DD strategies have been supported by Machine Learning algorithms, which have been developed with the standardized workflow analyzed in Section 3. Nevertheless, ML algorithms have suffered limitations when trying to reach real-time diagnosis because of some time-consuming manual stages. That is why, from 2015 until the present, Deep Learning end-to-end architectures have become the hot topic among the researchers.
- Concerning the analyzed workflow, although the ML algorithm selection, training and testing step (3rd step) looks like the most laborious step of the workflow, data acquisition and preprocessing steps can be considered to be the bottleneck of the complete development procedure. Therefore, DL approaches try to overcome the presented limitations. The workflow for this new solution removes the middle feature engineering step, building end-to-end Deep Neural Networks. This means that new features do not have to be selected manually by experts, instead they are computed automatically by adding hidden networks to the traditional Artificial Neural Networks (see Figure 12).
- The main drawback of DL approaches is not only the lack of interpretability, but also the lack of ability to explain specific phenomena. This is a disadvantage for diagnostic applications, where cause-effect relationships need to be identified, so that you can correct or reconfigure systems or even change designs. When an algorithm integrates all the steps by itself and does not give any information about the features or the sources of a faulty event, it is difficult to implement the feedback key element of Industrial AI explained in Section 1.
- At the same time, Section 4 shows evidence of the usage of data-driven FDD methodologies in fault diagnosis of electric drive subsystems and components. It collects applications from sectors such as railway, aviation, electric and hybrid vehicle, elevators, energy generation and electric grid.
- Another result obtained from the review is that DD active supervision strategies have been applied in most components of the electric drive generic architecture. However, according to the literature, the number of works dedicated to each subsystem depends strongly on the analyzed component. As it can be seen in Figure 13, sensors, electric machines and mechanical subsystem (bearings) are the ones with higher numbers of scientific papers.
  It can be said that solutions based on ML and DL are applied in the most complex and expensive components in terms of maintainability. For example, on the one hand, power electronics (inverters, rectifiers, etc.) or passive components (DC-link capacitors, filter inductors, braking resistors) are usually designed for the whole Life Cycle of the systems. Hence, maintenance actions are limited and faults should not appear. On the other hand, electric machines and bearings are components that need extensive maintenance. Thus, active supervision continues being an open topic for research.
- Moreover, most of the analyzed applications are developed with simulation or test-bench data sources. Little research shows solutions validated in real industrial environments with field data, as can be seen in Figure 14.
  As it has been mentioned in the introduction, one of the challenges of the Industrial AI is the lack of faulty event samples when facing real environment applications. Therefore, in most research, training AI models require supplementing real data with synthetic samples from simulations or test-benches. It is clear that real systems are designed not to fail, that is why faulty data are scarce. As a result, the dataset becomes unbalanced and it will cause great difficulties in conducting model training. In order to overcome this limitation, much of the research has been working on simulation and test-bench environments to acquire synthetic balanced datasets. Owing to the fact that it is much easier to develop a controlled failure scenario, which will not damage the operation of the real application.
- Furthermore, in electric drives, sensors used for monitoring have different sources and characteristics making the data acquisition heterogeneous and challenging. However, the most used variables are vibration for diagnosing mechanical failures and current or voltage for analysing electrical failures. Although the results of the review show this, it is worth mentioning that if the main objective is to reduce the Life Cycle Costs of the on-line CM strategy, the most efficient approach is to use the sensors already installed for control and protection tasks of the electric drive as data source for training the ML algorithms, and likewise not adding more sensors that increase the initial investment. Among the most used sensors are current, voltage, speed sensors, etc. It is true that at the level of fault diagnosis, sensors such as accelerometers, acoustic and thermal cameras can better identify the origin of the fault. However, their implementation is not economically feasible because it increases the costs of the electric drive that already suffers many market pressures for acquisition cost. In turn, adding these sensors increases the cost of maintenance. As a result, it is understood that these sensors have greater opportunities in the quality control of the manufacturing processes of the equipment and in the periodic inspection scheduled off-line.
- Finally, looking at the casuistry of failure modes addressed in the literature, it has been seen that most proposals are closely related to failures at the component level (bearing failures, shot-circuits or broken rotor bars, for example). However, there is a wider field of research related to failure modes at the subsystem or even, system level (control instabilities, undesired interaction between energy conversion steps, loss of comfort in the system user, etc.). These are cases that are difficult to emulate in simulation or laboratory environments because more than one subsystem interacts under given conditions. It is in these cases, at the application level, with complex industrial systems, which escapes expert knowledge, that Artificial Intelligence (both ML or DL) and the use of Big Data have the greatest potential compared to other techniques.

## 6. Future Research Directions

**Figure 15:**
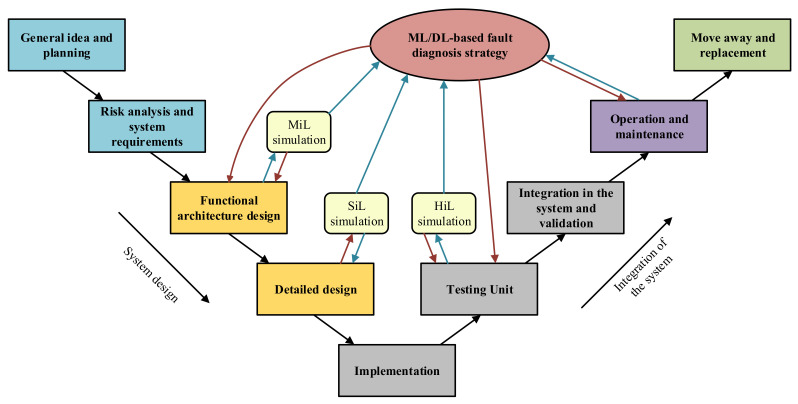
V model refresenting the Life Cycle of an industrial system.

Taking in mind this results, in next lines future challenges or investigation lines in which our research area will be focus on, are going to be explained:

1. Knowing that one of the main drawbacks of these data-driven fault diagnosis strategies, in real industrial applications, is the lack of faulty data (Unbalanced dataset), future research should work on dataset balancing techniques. An alternative to this challenge could be the synthetic data generation, via Hardware-in-the-Loop simulations, which should replicate as best as possible the real application under investigation, and also, should be compatible with the little field data available when training the ML algorithms. The second objective is to get closer to real applications building test-benches where different faulty scenarios can be forced, in order to generate efficient training datasets.
2. Another interesting future line is the perspective change when facing failure modes analysis. As mentioned before, failures are currently studied from a faulty component or subsystem point of view, and in most cases only faulty effects on the analyzed subsystems are considered. However, the interaction between subsystems and focusing on a complete system environment can be interesting. This way, new faulty events and improvements can be found.
3. At the same time, another interesting objective is to try boosting unsupervised ML or even DL strategies, owing to the fact that they are considered to be a hot topic at present. These approaches can overcome the laborious task of raw dataset labelling, which many times is really expensive and time consuming.
4. Finally, and concerning the last stage of the ML/DL standard workflow, the integration of the data-driven fault diagnosis approaches in the Life Cycle of any industrial equipment needs further research. The main idea in here is to think how this approach can be established to obtain as much profit as possible, not only in the operation and maintenance stage of the equipment, but also during the design and the integration phases. Figure 15 shows an example of integration of the Condition Monitoring strategy in the “V” shape Life Cycle of an industrial system. Each stage should benefit from the knowledge acquired thanks to massive data collection and analysis, and at the same time, design tools such as simulations should help training fault diagnosis algorithms

## 7. Conclusions

This review focused on data-driven active supervision strategies implemented in electric drives, which add value to the systems, improving their competitiveness in the market. Furthermore, this publication provides general guidelines to develop the Machine Learning workflow, which can be considered to be the brain of the complete active supervision approach. At the same time, the analyzed scientific documentation is evidence of the importance that data-driven methods are acquiring in the maintenance and health management tasks not only in electric traction systems, but also in the industry and in other sectors such as chemistry or economy. However, in order to settle these techniques problems such as unbalanced datasets, speed-up trainings or even DL algorithm understanding should be overcome. For that, solutions such as hybrid active supervision methods, semi-supervised algorithms, or data augmentation techniques could be interesting.

## Figures and Tables

**Table 1:** Cloud-based monitoring platforms for maintenance in electric traction sector.

Manufacturer	Application	Cyber-Physical Platform	Cyber-Space Tool
***Alstom*** [1,2,3]	Railway	HealthHub	Google Cloud
***Bombardier*** [3,4,5]	Railway	Optiflo	IBM Cloud
***Siemens Mobility*** [3,6]	Railway	Railigent	AWS
***Hitachi*** [7]	Railway	Lumada	Hitachi Smart Cloud
***CAF*** [8]	Railway	LeadMind	AWS
***KONE*** [9,10]	Vertical transport (Elevators)	KONE CARE	IBM Cloud
***Thyssenkrupp Elevator*** [11,12]	Vertical transport (Elevators)	MAX	Azure Cloud
***Otis*** [13]	Vertical transport (Elevators)	Otis ONE	Azure Cloud
***Siemens Gamesa*** [14]	Energy gen. (Wind power)	Pythia	-
***Vestas*** [15,16]	Energy gen. (Wind power)	-	TIBCO Spotfire

**Table 2:** Advantages and disadvantages of the active supervision methodologies.

	Advantages	Disadvantages	
***Signal-based***	Simple Fast	Expert knowledge required Symptoms similar in different faults Limited to detection/diagnosis tasks in ISO No attention to the dynamic behaviour of the system Expert knowledge required Many uncertainties difficult to be defined analytically Limited to diagnosis tasks in ISO High amount of data required Need for data storage platform High computational capacity
***Model-based***	Accurate in simple systems Easy to reach analytical redundancy	
***Data-driven***	Accurate if data available Extend ISO to Prognostics Assessment	

**Table 3:** Data-driven FDD application examples in DC-link subsystem.

Ref.	Faulty Event	Meas. Signal	Feat. Sel./Extr.	Algorithm	Data Source
[82]	GND fault	$v_{\mathrm{dc}}$, $i_{\mathrm{in}}$ and $i_{\mathrm{out}}$	*t*-domain	CCA	Test-bench	
[87]	C reduct.	$i_{\mathrm{in}}$ and $i_{\mathrm{out}}$	*t*-domain	ANN	Simu.	
[88]	Cap. ageing	$v_{\mathrm{in}}$ and $v_{\mathrm{dc}}$	*t*-domain	ANFIS	Simu.	
[89]	C reduct.	*i* and *v*	*t* and *f*-domain	SVR	Simu.	

**Table 4:** Data-driven FDD application examples in energy conversion subsystem.

Ref.	Faulty Event	Meas. Signal	Feat. Sel./Extr.	Algorithm	Data Source
[94]	OC	$i_{\mathrm{abc}}$	Wavelet	SVM	Simu.
[96]	OC	$v_{\mathrm{abc}}$	FFT and PCA	Bayessian Net.	Simu. and Test-bench
[100]	OC	$i_{\mathrm{abc}}$	-	Ensemble method	Simu.
[95]	OC and SC	$v_{\mathrm{abc}}$, $i_{\mathrm{abc}}$ and $T_{\mathrm{em}}$	*t*-domain	ANN	Simu.
[93]	OC	$v_{\mathrm{abc}}$, $i_{\mathrm{abc}}$ and $T_{\mathrm{em}}$	*t*-domain	ANN	Simu. and Test-bench
[101]	OC	$i_{\mathrm{abc}}$ and Clark *i*	*t* and *f*-domain	ANN	Test-bench
[102]	OC	$i_{\mathrm{abc}}$	Wavelet	SVM	Simu.
[103]	OC and SC	Gate sig.	-	KPCA	Test-bench
[97]	OC	$i_{\mathrm{abc}}$	Image converter	CNN	Simu.
[98]	OC	$i_{\mathrm{abc}}$	-	DWNN and DSVM	Simu.
[99]	OC	$i_{\mathrm{abc}}$ and Gate sig.	-	SRWNN and SVM	Simu. and Test-bench
[104]	OC	$i_{\mathrm{abc}}$	*t*-domain	Random Forest	Test-bench
[105]	OC	$v_{\mathrm{abc}}$	-	CNN	Simu.
[106]	OC	$i_{\mathrm{abc}}$	*t* and *f*-domain	ANN	Simu. and Test-bench
[107]	OC	$i_{\mathrm{abc}}$	FFT and ReliefF	ANN	Simu.

**Table 5:** Data-driven FDD application examples in electric machine subsystem.

Ref.	Faulty Event	Meas. Signal	Feat. Sel./Extr.	Algorithm	Data Source
[129]	Rotor and stator	Power	*t*-domain and LDA	SVM	Test-bench
[124]	Rotor and stator	Vibration	*f*-domain	DBN	Test-bench
[126]	Rotor and stator	Vibration	-	SAE-DNN	Test-bench
[130]	Brok. rotor bar	$i_{\mathrm{abc}}$	*t*, *f*-domain and Pearson Corr.	NB, *k*NN, AdaBoost, SVM and ANN	Test-bench
[120]	Rotor and stator	Vibration	DWT and GA	*k*NN, MLP and RBF	Test-bench
[131]	Broken rotor bar	$i_{\mathrm{abc}}$	*t*-domain	SVM, *k*NN and MLP	Test-bench
[132]	Rotor and stator	$i_{\mathrm{abc}}$	*t*-domain and DWT	ANN	Simu.
[118]	Winding inter-turn SC and rotor eccen.	$i_{\mathrm{abc}}$	*t*-domain and PCA	MLP	Test-bench
[133]	Rotor and stator	Vibration	-	CDFL-SVM	Test-bench
[50]	Turn-to-turn SC	$i_{\mathrm{abc}}$ and $v_{\mathrm{abc}}$	FFT and Fisher corr.	*k*NN, NB and SVM	Test-bench
[123]	Broken rotor bar and faulty rings	Thermal imaging	MoASoID imaging	*k*NN, *k*Means and ANN	Test-bench
[127]	Rotor and stator	Vibration and $i_{\mathrm{abc}}$	Wavelet	DCNN	Test-bench
[125]	Rotor and stator	Vibration	Wavelet	CNN (Transfer Learning)	Test-bench
[134]	Rotor and stator	Vibration	Wavelet	CNN	Test-bench
[135]	Rotor and stator	Vibration	*t*-domain, PCA, LDA and Fisher corr.	ANN	Test-bench
[122]	Broken rotor bar and faulty rings	Acoustic	*f*-domain	*k*NN and ANN	Test-bench
[136]	Stator winding SC	$i_{\mathrm{abc}}$	Clark transform	ANN	Test-bench
[119]	Stator winding SC and broken rotor bar	$i_{\mathrm{abc}}$	Wavelet and PCA	Decision Tree	Test-bench
[137]	Rotor eccentricity	$i_{\mathrm{abc}}$	FFT	ANN	Test-bench
[138]	Rotor	Vibration	PCA	CNN	Test-bench
[128]	Rotor	$i_{\mathrm{abc}}$	EMD	DNN	Test-bench
[139]	Rotor and stator	Vibration and $i_{\mathrm{abc}}$	*t*-domain and FFT	Decision Tree and *k*NN	Test-bench
[140]	Generic	$i_{\mathrm{abc}}$	-	LSTM-FCN	Test-bench
[141]	Rotor and stator	Vibration	-	DNN	Test-bench
[142]	Rotor and stator	$i_{\mathrm{abc}}$ and $v_{\mathrm{abc}}$	FFT	*k*Means	Simu.
[143]	Inter-turn SC	$i_{\mathrm{abc}}$	-	CNN	Simu.
[144]	Blocked air inlet	Thermal imaging	-	SVM and *k*NN	Test-bench

**Table 6:** Data-driven FDD application examples in mechanical subsystem (Bearings and Gearbox). Prat I.

Ref.	Faulty Event	Meas. Signal	Feat. Sel./Extr.	Algorithm	Data Source
[161]	Hole and scratch	$i_{\mathrm{abc}}$, ω and $v_{\mathrm{abc}}$	FFT	CNN, DT, RF, NB, SVM, *k*NN	Test-bench
[152]	Ball, inner and outer race	Vibration	FFT	CNN	Test-bench
[162]	Ball, inner and outer race	Vibration	Variational Mode Decomposition	DBN	Test-bench
[153]	Ball, inner and outer race	Vibration	STFT	CNN	Test-bench
[163]	Ball, inner and outer race	Vibration	Sparse Filtering	Softmax Regression	Test-bench
[151]	Generic faults	Vibration	*t*-domain, DA and CCA	MLP	Test-bench
[164]	Generic faults	Vibration	-	CNN	Real data
[165]	Generic faults	Thermal imaging	Wavelet	SVM	Test-bench
[166]	Generic faults	Leakage *i*	PCA	*k*NN	Test-bench
[149]	Generic faults	Vibration	Wavelet	SVM and ANN	Simu. and Test-bench
[167]	Ball, inner and outer race	Vibration	*t* and *f*-domain	ANN, Rule-based Method, SVM and *k*NN	Test-bench
[157]	Ball	Temperature and vibration	-	SVR	Test-bench
[168]	Ball, inner and outer race	Vibration	STFT	CNN and LSTM	Test-bench
[169]	Ball, inner and outer race	Vibration	FFT	DNN	Test-bench
[170]	Ball, inner and outer race	Audio	*t* and *f*-domain	*k*Means and *k*NN	Test-bench
[171]	Ball, inner and outer race	Vibration	EEMD, *t* and *f*-domain	WNN	Simu.
[154]	Ball, inner and outer race	Vibration	Wavelet and Autoencoder	ANN	Test-bench
[172]	Ball, inner and outer race	Vibration	-	Autoencoder and Softmax	Test-bench
[125]	Ball, inner and outer race	Vibration	Wavelet	CNN	Test-bench
[150]	Generic faults	$i_{\mathrm{abc}}$	Ambiguity plane	ANN	Test-bench
[173]	Ball, inner and outer race	Vibration	*t* and *f*-domain	Ensembl. SVM	Test-bench
[155]	Generic faults	Acoustic	STFT	CNN	Test-bench
[174]	Ball, inner and outer race	Acoustic	Wavelet	CNN	Test-bench
[175]	Ball, inner and outer race	Vibration	STFT	CNN	Test-bench
[158]	Ball, inner and outer race	Vibration	Wavelet	SVR	Test-bench
[176]	Inner race and outer race	Vibration	Autoencoder	DBN	Test-bench
[156]	Ball, inner and outer race	Thermal imaging	Autoencoder	CNN	Test-bench
[177]	Inner race and outer race	Thermal imaging	Wavelet and PCA	SVM, LDA and *k*NN	Test-bench
[48]	Generic faults	-	*t*-domain	Gaussian Regression	Real data
[175]	Ball, inner and outer race	-	STFT	CNN	Test-bench
[178]	Ball, inner and outer race	Vibration	*t*-domain	kNN	Test-bench
[179]	Tooth fracture and wear	$i_{\mathrm{abc}}$	*f*-domain	SVM	Test-bench
[160]	Tooth fracture and wear	Vibration	Wavelet	CNN, SVM and ANN	Test-bench
[180]	Tooth fracture, pitting and wear	Vibration and acoustic	*t* and *f*-domain	CNN	Test-bench
[181]	Inner and outer race	$i_{\mathrm{abc}}$	Genetic Alg.	*k*NN, DT and RF	Test-bench
[182]	Generic faults	$i_{\mathrm{abc}}$ and vibration	Autoencoder and LDA	ANN	Test-bench
[183]	Ball, inner and outer race	Vibration	FFT	Extreme Learning Machine	Test-bench

**Table 7:** Data-driven FDD application examples in sensors.

Ref.	Faulty Event	Meas. Signal	Feat. Sel./Extr.	Algorithm	Data Source
[190]	Bias and offset	$i_{\mathrm{abc}}$, ω and $v_{\mathrm{dc}}$	-	MPCA	Test-bench
[186]	Stuck, noise and offset	$i_{\mathrm{abc}}$, ω and $v_{\mathrm{dc}}$	-	Extreme Learning Machine	Simu. and Test-bench
[191]	Generic faults	$i_{\mathrm{abc}}$	*t*-domain	ANN	Simu. and Test-bench
[192]	Bias and offset	$i_{\mathrm{abc}}$, ω and $v_{\mathrm{dc}}$ and $T_{\mathrm{em}}$	CCA and KLD	CNN	Test-bench
[185]	Bias and offset	$i_{\mathrm{abc}}$, ω and $v_{\mathrm{dc}}$	PCA	SVM	Simu. and Test-bench
[184]	Bias and ramp	$i_{\mathrm{abc}}$	PCA and KLD	ANN	Test-bench
[193]	Ramp, stuck and offset	$i_{\mathrm{abc}}$, ω and $v_{\mathrm{dc}}$	-	DeepPCA	Test-bench
[187]	Generic faults	$i_{\mathrm{abc}}$ an ω	PCA	ANN and *k*NN	Simu.
[194]	Bias and offset	$i_{\mathrm{abc}}$, ω and $v_{\mathrm{dc}}$	-	PCA	Test-bench
[188]	Generic faults	Strain gauge	-	*k*NN	Real data
[189]	Generic faults	Temperature	*t*-domain	SVM	Simu.
[195]	Generic faults	$v_{\mathrm{abc}}$ and $v_{\mathrm{dc}}$	-	Extreme Learning Machine	Test-bench
[196]	Stuck, noise, gain and offset	$i_{\mathrm{abc}}$, ω and $v_{\mathrm{dc}}$	*t* and *f*-domain	Extreme Learning Machine	Simu. and Test-bench

## Data Availability

Not applicable.

## Abbreviations

The following abbreviations are used in this article:

FDD	Fault detection and diagnosis
ML	Machine Learning
DL	Deep Learning
CM	Condition Monitoring
AI	Artificial Intelligence
BD	Big Data
IoT	Internet of Things
CC	Cloud Computing
AWS	Amazon Web Services
TCU	Traction Control Unit
LCC	Life Cycle Cost
SB	Signal-based
MB	Model-based
DD	Data-driven
FFT	Fast Fourier Transform
STFT	Short Time Fourier Transform
PCA	Principal Component Analysis
RUL	Remaining Useful Life
ESS	Energy Storage Systems
ANN	Artificial Neural Network
SVM	Support Vector Machine
CNN	Convolutional Neural network
MLP	Multi-Layer Perceptron
DNN	Deep Neural Network
